# Correction: The immunology of hypertension

**DOI:** 10.1084/jem.2017177301022018c

**Published:** 2018-02-05

**Authors:** Allison E. Norlander, Meena S. Madhur, David G. Harrison

Vol. 215, No. 1, January 2018. https://doi.org/10.1084/jem.20171773

The authors regret that a typographical error appeared in the first version of their paper, where IL-1 was cited in place of IL-17A. The affected paragraph appears below with the corrected text:

Although the majority of T cell receptors are composed of α/β chains, a small percentage possess γ/δ receptors, and recent evidence suggests that these also contribute to hypertension. Caillon et al. (2017) recently showed that γ/δ T cells are important in hypertension, and that mice lacking these cells exhibit a markedly blunted rise in blood pressure and preserved endothelial function in response to Ang II infusion. γ/δ T cell–deficient mice also exhibited fewer activated CD4^+^ T cells expressing the marker CD69 in the spleen and mesenteric arteries, suggesting that γ/δ T cells might have an initiating role in hypertension. Likewise, antibody clearance of γ/δ T cells reduced the hypertensive response to Ang II. The precise mechanisms by which these cells promote hypertension remain to be defined, but these cells are also major sources of IL-17A, which has prohypertensive actions in both the kidney and vasculature (Saleh et al., 2016).

The online HTML and PDF versions of this paper have been corrected. The error remains only in the print version.

